# Anesthetic Challenges in a Patient With an Expanding Recurrent Mycotic Pseudoaneurysm of the Right Brachiocephalic Artery: A Case Report and Literature Review

**DOI:** 10.7759/cureus.55372

**Published:** 2024-03-02

**Authors:** Muhammad Jaffar Khan, Temur Baykuziyev, Tarek Tageldin, Abdulatif Albasha

**Affiliations:** 1 Anesthesiology, ICU and Perioperative Medicine, Hamad Medical Corporation, Doha, QAT

**Keywords:** tracheal compression, airway obstruction, brachiocephalic artery, pseudoaneurysm, mycotic

## Abstract

Airway obstruction requires urgent intervention. When dealing with the right brachiocephalic artery mycotic pseudoaneurysms, the risk of rupture and massive hemorrhage adds greater urgency to the management. Furthermore, tracheal compression presents difficulties during airway management. This report highlights the airway and anesthetic challenges encountered during the procedure and emphasizes the importance of tailored intervention for optimal patient care.

We describe the clinical case of a 38-year-old male patient who presented with a large recurrent right brachiocephalic artery pseudoaneurysm associated with tracheal compression. The patient required urgent surgical intervention due to the pseudoaneurysm's enlargement and progressive respiratory distress. Awake fiber-optic intubation was not feasible. A cardiopulmonary bypass was kept on standby in the event of failed intubation and ventilation, or circulatory collapse.

Endotracheal intubation was performed successfully using a video-laryngoscopy. After successful surgical repair of the pseudoaneurysm, the patient was transferred to ICU where he was extubated 48 hours post-surgery, following treatment with methylprednisolone for edematous aryepiglottic folds identified during video-laryngoscopy.

Overall, this case emphasizes the importance of early diagnosis, prompt surgical intervention, and effective teamwork in managing rare and potentially life-threatening conditions like mycotic pseudoaneurysms. It also highlights the critical role of anesthesiologists in providing optimal perioperative care, ensuring hemodynamic stability, managing airway challenges, and facilitating successful surgical outcomes. In our work, we also provide a summary of the reported similar cases.

## Introduction

Mycotic pseudoaneurysms of the brachiocephalic artery are rare but potentially life-threatening conditions characterized by infected arterial dilatation. They typically result from bacterial or fungal dissemination to arterial walls. Like numerous other aneurysms, brachiocephalic artery aneurysms present significant risks such as rupture (4.1-11%) and local compression. Additionally, they carry the potential for catastrophic brain embolization and stroke. Prompt surgical intervention is crucial to prevent rupture, and associated complications [1,2].

During the induction of anesthesia, airway collapse may occur following the administration of induction drugs due to skeletal muscle relaxation. This situation can lead to sudden and significant hypoxia if prompt establishment of airway patency is not achieved through endotracheal intubation. If severe compression of the trachea occurs, intubation can become exceptionally challenging. If the patient is cooperative, an awake fiber-optic intubation could be considered the preferred method for airway management. Additionally, a strategy involving awake peripheral cannulation and the initiation of cardiopulmonary bypass can be devised.

Herein, we describe the anesthetic approach to a patient requiring urgent surgical repair for a recurrent mycotic pseudoaneurysm of the right brachiocephalic artery causing tracheal compression and airway obstruction.

## Case presentation

**Figure 1:**
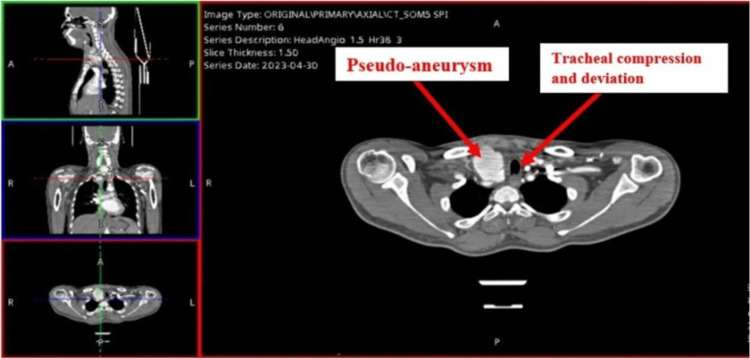
CT-scan showing pseudoaneurysm compressing trachea (axial view on right).

**Figure 2:**
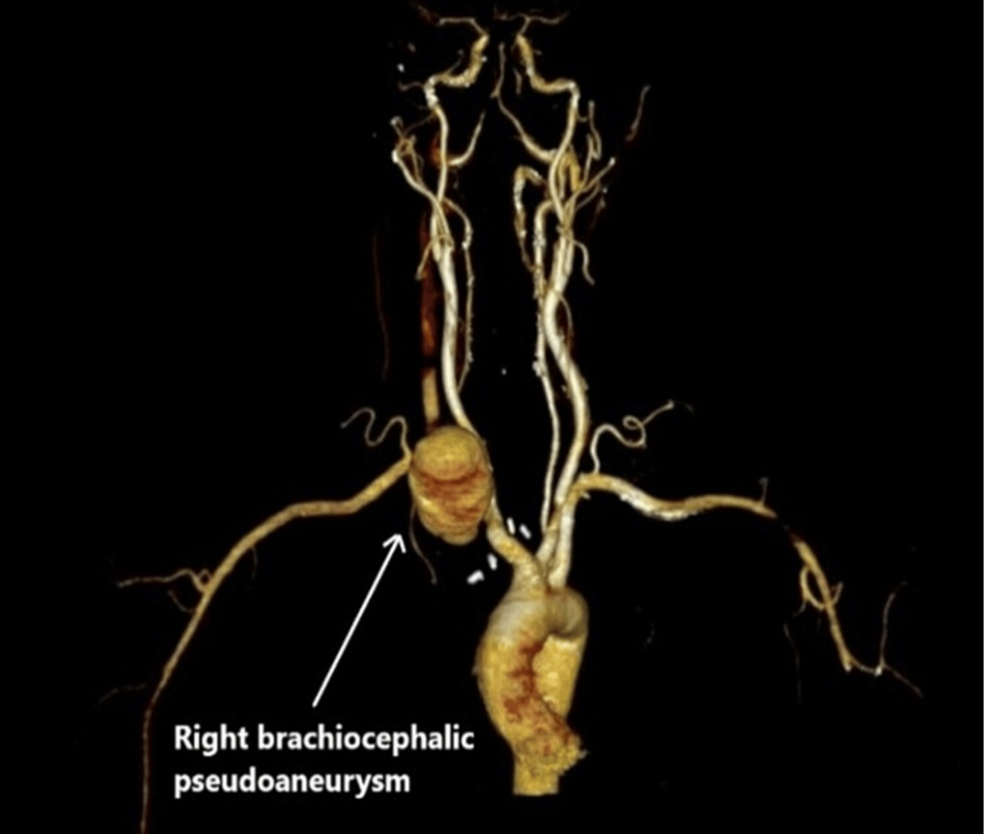
CT-angiography demonstrating right brachiocephalic pseudoaneurysm.

A 38-year-old male patient presented to the emergency department with a history of right neck swelling for three days associated with neck pain. He has a past surgical history of mini sternotomy and open repair of pseudoaneurysm of the right subclavian artery eight months ago. Additionally, the patient has a medical history of poorly controlled hypertension. Initial workup showed WBC of 18.8x103/uL, CRP 181 mg/L, ESR 70 mm/hr. CT chest revealed a 51 x 31 mm enlarged pseudo-aneurysm arising from the right brachiocephalic artery associated with compression and displacement of the trachea (Figure 1, 2).

**Figure 3:**
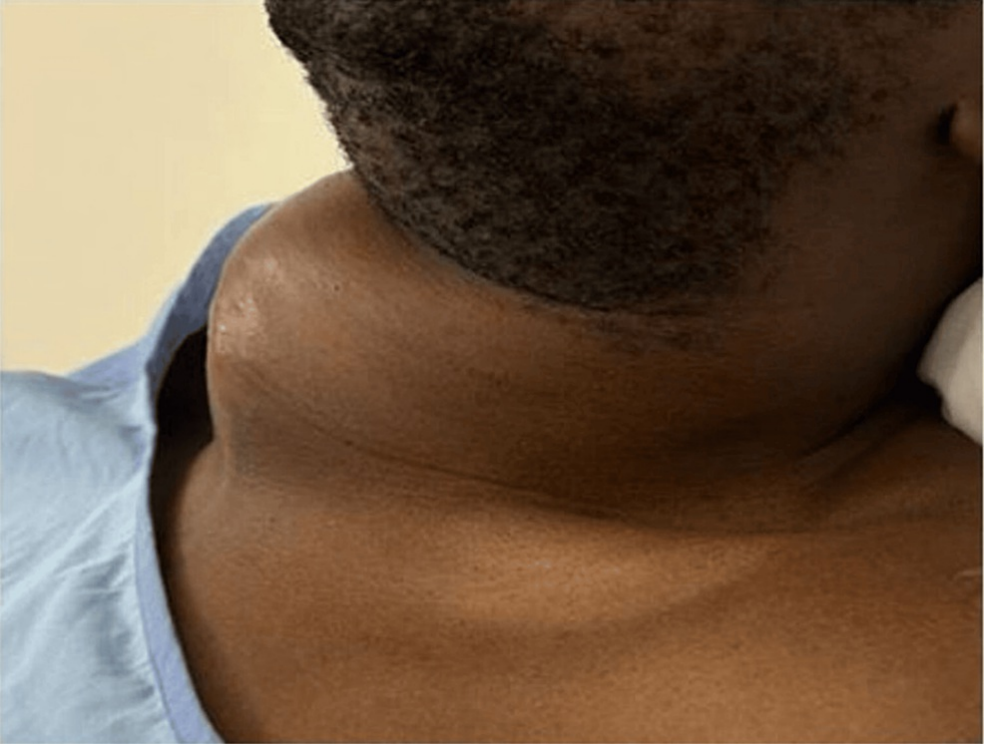
Pulsatile neck mass lateral view.

The patient was started on empiric antibiotics (piperacillin/tazobactam and vancomycin). A positron emission tomography-computed tomography (PET-CT) scan was planned to rule out endovascular infection. However, the patient developed a rapidly expanding, pulsatile neck mass (Figure 3) during the admission which was associated with compression symptoms and deemed a high risk for impending respiratory failure. It was decided to take him to the operating theater for urgent surgical intervention. Given the urgency of the situation and due to the presence of a quickly growing, pulsatile neck mass and the inability to establish a surgical airway, the decision was made to proceed with general anesthesia as the only viable option. Awake fiberoptic intubation was not feasible in this case as the patient was uncooperative and in respiratory distress.

Anesthetic technique

American Society of Anesthesiologists (ASA) standard monitors were applied, followed by the insertion of a left radial artery cannula under local anesthesia for continuous blood pressure monitoring. Cerebral oximetry was also used to assess cerebral perfusion. The femoral artery and vein were prepped in anticipation of promptly instituting cardiopulmonary bypass in the event of failed intubation and ventilation, or circulatory collapse. It was decided to proceed with intravenous induction of general anesthesia, to avoid the risk of coughing and/or laryngospasm associated with awake fiberoptic intubation. Preoxygenation was performed by administering 100% oxygen at a rate of 15 liters per minute through an anesthesia circle circuit and a face mask with a proper seal until the end-tidal oxygen (ETO2) concentration reached 90%.

**Figure 4:**
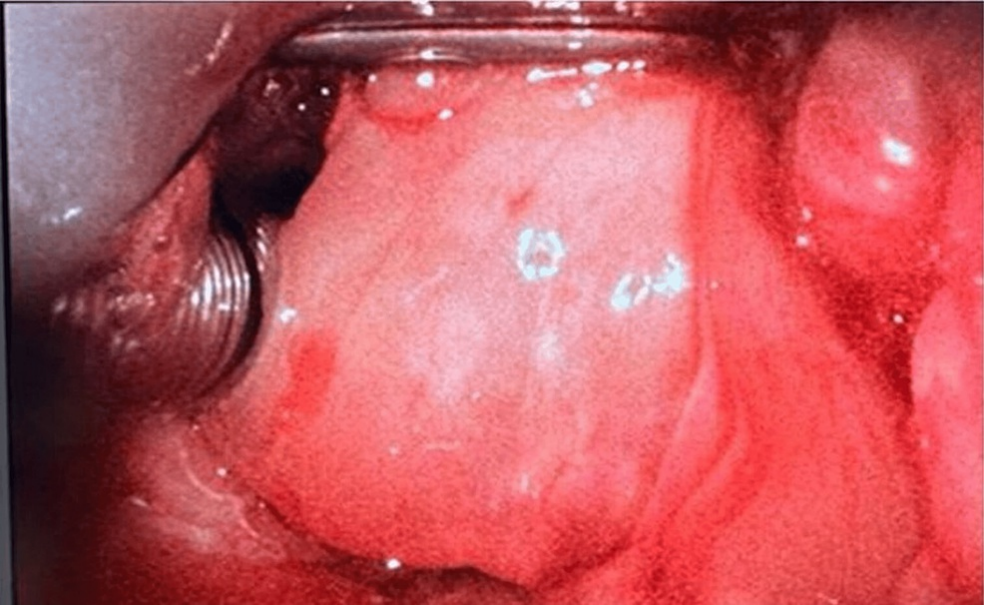
Laryngeal view demonstrated via C-MAC D-Blade® video-laryngoscope

General anesthesia was induced by intravenous administration of medications in the following sequence: 2 mg of midazolam, 200 mcg of fentanyl, and 150 mg of propofol. Following confirmation of adequate mask ventilation, 100 mg of rocuronium was administered intravenously. Endotracheal intubation was achieved using a video-laryngoscope (C-MAC D-Blade®, Karl Storz, Tuttlingen, Germany) and an armored tube (size 7.0 mm ID) over a stylet to facilitate the passing of the tube beyond the tracheal compression. The laryngeal view was edematous and displaced to the left side, posing a challenge for intubation (Figure 4). However, with careful neck manipulations, successful intubation was achieved without oxygen desaturation. A flexible bronchoscope was utilized to assess the trachea post-intubation. Anesthesia was then maintained by sevoflurane inhalation and remifentanil intravenous infusion.

After intubation, a femoral central line was placed for administering vasopressors and two 16G peripheral lines were inserted. Intraoperatively, FloTrac® (Edwards Lifesciences Corp, Irvine, CA, USA) continuous cardiac output monitor tracked stroke volume variation, systemic vascular resistance, cardiac output, and cardiac index.

Intraoperative course

After the induction of anesthesia, an angiogram of the right brachiocephalic artery was performed to assess the extent of the pseudoaneurysm. A destination sheath was inserted through the femoral artery and a sheath with an inflatable balloon was positioned in the proximal part of the right brachiocephalic artery. This was done to provide control in case of bleeding or rupture during the subsequent surgical repair.

Following the angiogram and positioning of the sheath, the surgical team proceeded with sternotomy and open repair of the right brachiocephalic artery pseudoaneurysm. During the dissection of the right common carotid artery, a sudden rupture of the pseudoaneurysm occurred, resulting in bleeding. Despite the presence of the balloon in the proximal part of the right brachiocephalic artery, it was unable to control the bleeding effectively. Consequently, the balloon was removed, and the surgical team swiftly responded by applying manual pressure to achieve control over the bleeding site. Through this immediate intervention, hemostasis was successfully attained, ensuring the cessation of bleeding.

Cardiopulmonary bypass remained on standby; in case of an emergent need for circulatory support. However, the surgical team successfully managed the bleeding using manual control techniques. Additionally, the patient received 6000 ml of crystalloid fluids, 750 ml of colloid fluids, 3 units (858 ml) of packed red blood cells, and 1400 ml of autologous blood via Cell Saver (Haemonetics, Boston, MA, USA) and noradrenaline infusion during the surgery. The complex surgery lasted approximately six hours, ensuring successful vascular reconstruction, hemostasis, and placement of bypass graft.

Postoperative management

The patient was transferred to the surgical intensive care unit (SICU) after the surgery; and kept intubated, sedated, and mechanically ventilated. Airway edema was detected on video laryngoscopy. A dose of 20 mg of methylprednisolone was administered intravenously every six hours to reduce the edema. The patient underwent successful extubation approximately 48 hours after the surgery, following assessment of the airway using video-laryngoscopy, ensuring reduced edema. Additionally, a positive cuff leak test, normal arterial blood gas analysis, and stable clinical condition of the patient were ensured before extubation. Methylprednisolone was continued for the next 24 hours. On the fifth postoperative day, with satisfactory recovery, the patient was discharged to the general ward.

## Discussion

Brachiocephalic artery or innominate artery aneurysms are relatively rare with an incidence of 2 to 5 percent of all aneurysms affecting the aortic arch vessels and less than 1 percent of peripheral artery aneurysms in general [1,2]. The primary cause of innominate artery aneurysms is degenerative disease or atherosclerosis, which is responsible for over 50 percent of true innominate artery aneurysms. Other less common causes include syphilis, tuberculosis, Kawasaki disease, Takayasu arteritis, Behcet disease, and connective tissue disorders such as Marfan syndrome and Ehlers-Danlos syndrome. Trauma can also lead to innominate artery aneurysms, although most cases in this category are pseudoaneurysms. Mycotic (infected) innominate aneurysms are rare [3]. The arteries frequently affected by mycotic pseudoaneurysms include the aorta, peripheral arteries, cerebral arteries, and visceral arteries. Approximately 13.3% of mycotic pseudoaneurysms arise from infection of pre-existing aneurysms, with bacterial organisms, including Gram-positive cocci (predominantly *Staphylococcus aureus*), accounting for 55%, and *Salmonella *accounting for 30-40%.

Most brachiocephalic artery aneurysms are found incidentally, while about 25 percent of the cases present with symptoms. Symptoms can vary and include pain due to expansion or rupture, ischemic symptoms affecting the brain or upper extremities, brachial plexus compression, hoarseness, dyspnea due to tracheal compression, Horner syndrome, hemoptysis, and rare cases of hemodynamic collapse from aneurysm rupture occurring in approximately 10 percent of patients [2-4]. CT angiography is the preferred and reliable imaging modality for assessing innominate artery aneurysms. It offers precise information on aneurysm size, thrombus presence, and the anatomical relationship between the aneurysm and adjacent structures. Treatment is recommended for symptomatic innominate artery aneurysms, as well as those with thrombus, which poses a potential risk of thromboembolic stroke or limb-threatening ischemia. Although open surgical repair is still considered the standard approach for treating brachiocephalic artery aneurysms, it is associated with notable morbidity and mortality. This is primarily due to the requirement of a median sternotomy and cardiopulmonary bypass. Mycotic pseudoaneurysm is a severe clinical condition that carries substantial morbidity and mortality risks. The recommended treatment approach involves a combination of antibiotic therapy and extensive surgical debridement of the infected tissue, along with vascular reconstruction as necessary.

Anesthesia/airway challenges

Tracheobronchial compression by the vascular anomaly poses great airway and anesthetic challenges. Acute airway obstruction is associated with an increased risk of perioperative cardiorespiratory collapse [5]. Compression typically arises due to the close anatomical proximity between the aortic arch vessels and the trachea, as well as the left main stem bronchus. Acute airway obstruction caused by the aneurysm of the brachiocephalic artery has been reported in the literature [6].

Various methods are available for managing the airway with tracheal compression, including awake fiberoptic intubation above or below the narrowed area with the patient spontaneously breathing; General anesthesia with endotracheal intubation; General anesthesia using patient’s natural airway or spontaneous ventilation; employing high-frequency ventilation; utilizing cardiopulmonary bypass; and inserting an endotracheal stent. Each approach carries its own set of advantages and disadvantages.

Awake fiberoptic intubation is widely recognized as a safe and effective method for managing difficult airways. However, in certain situations, it may be necessary to reconsider the initial plan and opt for an alternative airway-securing technique. We considered the possibility of performing awake fibreoptic intubation with or without sedation to maintain the patient's spontaneous breathing. However, our patient was uncooperative due to respiratory distress. Also, we recognized the potential complications arising from inadequate topicalization and an uncooperative patient, which could result in loss of vision during the procedure, coughing, and/or laryngospasm. Such complications could increase the transmural pressure of the pseudoaneurysm and potentially lead to life-threatening rupture or further compression of the trachea.

A flexible bronchoscope facilitates the assessment of airway portions and airway wall properties including the endoluminal compression by the aneurysm and effective clearing of secretions. Numerous reports highlight the importance of intraoperative bronchoscopic monitoring of airway decompression and its effectiveness in these cases [6,7]. Therefore, we decided to choose a reinforced tube (size 7.0 mm) with a soft tip to reduce the likelihood of tracheal or aneurysm rupture and assessed the airway with a Flexible bronchoscope post-intubation.

Choice of airway equipment should be wisely instituted especially in patients with large aneurysms requiring lung isolation. Double-lumen tubes should be avoided due to anticipated difficulty in placement and also the potential risk for rupture of aneurysm. However, bronchial blockers can be utilized for lung isolation if necessary. In our case, lung isolation was not required.

It is imperative to prepare alternative options in case endotracheal intubation fails to maintain oxygenation and ventilatory demands. Therefore, before inducing anesthesia, percutaneous cardiopulmonary support should be readily available [8,9]. In our patient, the femoral artery and vein were prepped in anticipation of promptly instituting cardiopulmonary bypass in the event of failed intubation and ventilation, or circulatory collapse.

Effective blood pressure management is crucial in tracheobronchial-vascular compression syndrome. Hypertension not only increases the risk of aneurysm rupture but also elevates the pressure exerted by the aneurysm on the trachea. Therefore, it enhances the likelihood of further airway obstruction. Uncontrolled hypertension in our patient might have contributed to the rapid and progressive expansion of the pseudoaneurysm. The control of the blood pressure immediately was of paramount importance, which we did by using labetalol preoperatively.

Tracheomalacia can occasionally be linked to congenital or acquired abnormalities of the aortic arch. The acquired form of tracheomalacia is usually a result of prolonged external compression from a mediastinal mass. However, there have been rare cases reported in the literature where tracheomalacia was caused by a chronic aortic arch aneurysm [10]. A fiberoptic bronchoscope is a useful tool to diagnose tracheomalacia and also expandable metallic stent can be deployed.

This case is characterized by several challenges, including a difficult airway due to neck mass compression and airway edema, as well as an intraoperative rupture of the pseudoaneurysm (Table 1). However, with meticulous planning, proper monitoring, and prompt interventions, these challenges were successfully overcome, ensuring the patient's safety and optimal surgical outcomes. 

**Table 1:** Summary of anesthetic concerns in mycotic pseudoaneurysm of the brachiocephalic artery.

Preoperative
Extrinsic compression of structures (trachea, major vessels, right heart): Review all imaging with a multidisciplinary team.
Underlying infection: Treat with antibiotics.
Surgical vs endovascular repair vs conservative.
Risk of rupture: Close hemodynamic monitoring and blood pressure control.
Intraoperative
Anticipated difficult airway: Consider awake fiberoptic intubation or intravenous/inhalational induction with cardiopulmonary bypass standby.
Monitoring: For invasive arterial lines and central lines, consider cardiac output monitoring.
Hemodynamic: Compromised cardiac output due to right ventricular compression or other major vessels (consider intraoperative transesophageal echocardiography).
Massive bleeding: Large bore cannula, blood products to be readily available, cell salvage, or cardiopulmonary bypass (CPB).
Cerebral ischemia: Neuromonitoring, cerebral oximetry.
Postoperative
Airway: Video laryngoscopy or fiberoptic bronchoscopy to rule out airway edema or tracheomalacia.
Circulation: Close hemodynamic monitoring.
Cerebral: Neuromonitoring/CT head.
Infection: Continuous antimicrobial coverage of mycotic aneurysm.

This case report contributes to the existing literature (Table 2) by documenting the airway and anesthetic management; and surgical techniques employed in the repair of a brachiocephalic artery mycotic pseudoaneurysm. Sharing such experiences helps enhance our understanding of these complex cases and guides future clinical decision-making for similar presentations.

**Table 2:** Review of case reports on airway management in aneurysms

Authors and publication year	Patient’s age and gender	Diagnosis CT findings	Airway or tracheal compression	Anesthesia or airway management	Surgical intervention	Outcome
Kumar A et al., 2016 [7]	42 years old, male	Large fusiform aneurysm of the arch of aorta and proximal descending thoracic aorta, distal to the left common carotid artery with involvement of origin of the left subclavian artery	Compression and narrowing of the trachea were seen with displacement of the trachea and esophagus toward the right, mild compression of the left main bronchus	General anesthesia induction with endotracheal intubation, followed by fiberoptic bronchoscopy to show the level of compression	Open repair on cardiopulmonary bypass	Extubated on postoperative day 6
Jung HJ et al., 2009 [8]	50 years old, male	Aneurysm of the innominate artery with a large thrombus from rupture of the medial wall of the proximal innominate artery	Compression of trachea	Emergency intubation with midazolam (5 mg intravenous), followed by anesthetic induction with muscle relaxation in the operation room	Open repair on cardiopulmonary bypass	Uneventful recovery
Dunn SA et al., 2019 [9]	Not reported	Pseudoaneurysm of the ascending aorta caused by mycotic infection of the aortic graft at the area of anastomosis from a type A dissection repair three years before	Tracheal deviation and compression of both mainstem bronchus	Awake femoral veno-arterial cardiopulmonary bypass followed by general anesthesia induction and intubation	Open repair	Not reported
Nishiwaki K et al., 1990 [11]	55 years old, male	Aneurysm of the ascending aorta	Compression from the anterior tracheal wall from 2.5 cm to the vocal cords to 8.5 cm below	General anesthesia with armored endotracheal tube size 6 mm, followed by bronchoscopy guided tube advancement beyond stenosis with induced hypotension	Open repair of aneurysm	Uneventful recovery
Gorman RB et al., 1993 [12]	77 years old, male	Dissecting thoracoabdominal aneurysm	No compression	General anesthesia induction followed by single lumen endotracheal tube 8.5 mm, left lung isolation with Fogarty catheter, post-intubation bronchoscopy revealed lower one-third trachea 50% narrowed and right mainstem bronchus completely occluded	Open left thoracotomy while on cardiopulmonary bypass	Extended postoperative course
Koomen E et al., 2007 [13]	70 years old, male	Aneurysmal dilation of the middle part of the descending thoracic aorta	Compression of the trachea and right main bronchus	General anesthesia with single lumen endotracheal tube size 9.0 mm with intermittent positive-pressure ventilation, supplemented by high-frequency jet ventilation and left-aorto-femoral bypass for surgical access	Left thoracotomy and open repair of aneurysm	Weaned off from the mechanical ventilator on postoperative day 2
Constenla I et al., 2012 [14]	63 years old, male	Aneurysm of the innominate artery (IA) (brachiocephalic trunk) with a maximum diameter of 4.5 cm	Tracheal compression	General anesthesia and fiberoptic bronchoscopy were used to achieve orotracheal intubation	Bypass from the ascending aorta to both common carotid arteries using a Dacron graft	Immediately extubated postoperative
Mundada SD et al., 2016 [15]	36 years old, female	Pseudoaneurysm arising from the right lateral wall of the ascending aorta	No compression on the trachea	General anesthesia induction with a 7.5 mm cuff tube under direct laryngoscopy, followed by femoral veno-arterial cardiopulmonary bypass	Open repair on cardiopulmonary bypass	Extubated on postoperative day 1
Arora V et al., 2021 [16]	34 years old, male	Pseudoaneurysm arising from the junction of the right common carotid artery and subclavian artery	Tracheal compression	Awake femoral veno-arterial cardiopulmonary bypass, followed by general anesthesia induction and intubation; fiberoptic bronchoscopy to guide the tube beyond the compression point	Open repair	Extubated on postoperative day 1
Das D et al., 2022 [17]	45 years old, female	Pseudoaneurysm of right common carotid artery	Localized mass effect on the thyroid, larynx, trachea, and esophagus with a shift towards the left was detected	Emergency airway due to ruptured aneurysm: general anesthesia with endotracheal intubation, followed by immediate femoral veno-arterial cardiopulmonary bypass	Open repair with Gortex graft	Extubated on postoperative day 2
Suda Y et al., 2022 [18]	83 years old, female	Impending rupture of the ascending thoracic aortic aneurysm and esophageal stenosis with significant amounts of food residues in the upper thoracic esophagus	Thoracic aortic aneurysm compressed the pulmonary artery and left bronchi	Awake endotracheal intubation was performed using a McGrath MAC® video laryngoscope with light sedation, followed by general anesthesia	Open repair of thoracic aortic aneurysm	Extubated on postoperative day 4
Montane-Muntane M et al., 2023 [19]	16 years old, male	Ascending aortic aneurysm (94 mm × 78 mm) that extended along 10 cm starting at 25 mm from the aortic ring	Extrinsic compression of the distal trachea	Step 1: General anesthetic (GA) induction, endotracheal intubation (7.5 mm) with direct laryngoscopy (failed ventilation); Step 2: Laryngeal mask airway insertion followed by fiberoptic intubation (7.0 mm endotracheal tube)-extremely difficult ventilation; Step 3: Immediate femoral arterial-venous bypass	Ascending aorta and arch replacement with Hemashield tube graft and reimplantation of the brachiocephalic trunk and left common carotid using a bifurcated prosthesis	Extubated on postoperative day 1
Khan MJ et al., 2023 [20]	37 years old, male	Pseudoaneurysm in the proximal right subclavian artery (5 x 4 x 5 cm)	Significant tracheal narrowing (>70%)	Inhalational induction with airway topicalization allows spontaneous breathing and C-MAC video laryngoscopy for endotracheal intubation, followed by intravenous induction and fiberoptic bronchoscopy to guide the tube beyond the stenosed area with extracorporeal membrane oxygenation back-up	Repair of the pseudoaneurysm and bovine patch graft	Extubated on postoperative day 2

## Conclusions

This case highlights the significance of timely diagnosis, prompt surgical treatment, and efficient teamwork in addressing rare and potentially life-threatening conditions like mycotic pseudoaneurysms. It also emphasizes the critical role played by anesthesiologists in delivering optimal perioperative care, ensuring stable hemodynamics, managing airway difficulties, and enabling successful surgical outcomes.
